# Rates of asymptomatic respiratory virus infection across age groups

**DOI:** 10.1017/S0950268819000505

**Published:** 2019-04-15

**Authors:** M. Galanti, R. Birger, M. Ud-Dean, I. Filip, H. Morita, D. Comito, S. Anthony, G. A. Freyer, S. Ibrahim, B. Lane, N. Matienzo, C. Ligon, R. Rabadan, A. Shittu, E. Tagne, J. Shaman

**Affiliations:** 1 Department of Environmental Health Sciences, Mailman School of Public Health, Columbia University, 722 West 168th Street, New York, NY 10032, USA; 2 Department of Systems Biology, Columbia University, 1130 St. Nicholas Ave, ICRC Bldg. 8th Floor New York, NY 10032, USA; 3 Department of Epidemiology, Columbia University, 722 W 168th St, New York, NY 10032, USA

## Abstract

Respiratory viral infections are a leading cause of disease worldwide. A variety of respiratory viruses produce infections in humans with effects ranging from asymptomatic to life-treathening. Standard surveillance systems typically only target severe infections (ED outpatients, hospitalisations, deaths) and fail to track asymptomatic or mild infections. Here we performed a large-scale community study across multiple age groups to assess the pathogenicity of 18 respiratory viruses. We enrolled 214 individuals at multiple New York City locations and tested weekly for respiratory viral pathogens, irrespective of symptom status, from fall 2016 to spring 2018. We combined these test results with participant-provided daily records of cold and flu symptoms and used this information to characterise symptom severity by virus and age category. Asymptomatic infection rates exceeded 70% for most viruses, excepting influenza and human metapneumovirus, which produced significantly more severe outcomes. Symptoms were negatively associated with infection frequency, with children displaying the lowest score among age groups. Upper respiratory manifestations were most common for all viruses, whereas systemic effects were less typical. These findings indicate a high burden of asymptomatic respiratory virus infection exists in the general population.

## Introduction

Respiratory viral infections are a leading cause of disease worldwide, affecting all age groups and representing a serious threat to human health, particularly for infants, older adults and the immunocompromised. The effects of infection on individuals can vary considerably and include completely asymptomatic manifestations, mild upper respiratory effects and severe symptoms requiring hospitalisation.

The epidemiology of respiratory viral infections is generally analysed through passive symptom-based surveillance. When studying the burden of infection, many observational studies focus on severe outcomes, such as cardiac and pulmonary complications in hospitalised patients [1–4], or the role of respiratory viruses in the exacerbation of pre-existing respiratory conditions [5–7]. Additionally, community-based longitudinal studies have generally been restricted to young children or households and involve the sampling of specimens to identify viruses *after* an index symptomatic episode occurs [8–10].

Investigation of the prevalence and effects of respiratory viral infections in the broader population, not just among individuals seeking medical attention, is needed to more fully understand the burden of these infections within the community and to develop adequate preventive measures against these pathogens. In particular, the proportion of the population that is infected and yet does not develop symptoms must be determined to better quantify transmission risk, forecast future disease incidence and pathogen spread, and support public health response efforts. Here, we document rates of asymptomatic respiratory virus infection through a large-scale community study across multiple age groups. We use data from a cohort of individuals who were tested weekly for respiratory viruses irrespective of symptom status. We combine these test results with participant-provided daily records of cold and flu symptoms and use this information to characterise symptom outcomes by virus and age category.

## Methods

### Cohort composition and survey

We enrolled 214 individuals from multiple locations in Manhattan borough, New York City. We refer to the participants as *healthy* as they were enrolled from the general population, as opposed to individuals seeking clinical care. The cohort included children attending two daycares, along with their siblings and their parents, teenagers and teachers from a high school, adults working at two emergency departments (a paediatric ER and an adult ER), and adults working at a university medical centre. The study period spanned 2 years from October 2016 to April 2018 with some individuals enrolled for a single cold and flu season (October–April) and others for the entire period. All individuals from the selected facilities who were willing to participate were enrolled in the study. Enrolled individuals were asked to complete a baseline survey and provide two nasopharyngeal swab samples (one from each nostril). Following this preliminary step, two nasopharyngeal samples were again collected weekly from each participant irrespective of symptoms. The baseline questionnaire completed at the time of enrolment included information on ethnicity, general health, daily habits, travel history and household structure. For the entire duration of the study, participants provided a daily report rating nine respiratory illness-related symptoms (fever, chills, muscle pain, watery eyes, runny nose, sneezing, sore throat, cough, chest pain), which were recorded on a Likert scale (0 = none, 1 = mild, 2 = moderate, 3 = severe). Participants were also asked to note if they had sought medical attention, stayed home or taken influenza-like illness (ILI)-related medications as a consequence of their listed symptoms. Parents provided consent, baseline questionnaire answers and the daily survey information for their enrolled children. A total daily score was generated for each participant by summing the scores of the individual symptoms (total daily score ranges from 0 to 27). Details on the participants are summarised in Table 1.

**Table 1.** Demographics of the study cohort

	Children	Parents	Teenagers	Teachers	Peds ED	Adult ED	Medical centre	All cohorts
	*n* %	*n* %	*n* %	*n* %	*n* %	*n* %	*n* %	*n* %
Enrolled	35 (16.4)	20 (9.3)	42 (19.6)	15 (7.0)	22 (10.3)	11 (5.2)	69 (32.2)	214 (100)
Total samples	1016 (24.1)	524(12.4)	361 (8.6)	248 (5.9)	537 (12.7)	103 (2.4)	1426 (33.8)	4215 (100)
Year (% per cohort)								
One	8 (22.9)	8 (40.0)	33 (78.6)	7 (46.6)	11 (50.0)	0 (0)	15 (21.7)	82 (38.3)
Two	10 (28.6)	6 (30.0)	9 (21.4)	1 (6.7)	2 (9.1)	11 (100)	41 (59.4)	80 (37.4)
Both	17 (48.5)	6 (30.0)	0 (0)	7 (46.6)	9 (40.9)	0 (0)	13 (18.9)	52 (24.3)
Gender (% per cohort)								
Male	17 (48.6)	3 (15.0)	27 (64.3)	8 (53.3)	7 (31.8)	4 (36.4)	27(39.1)	93 (43.5)
Female	18 (51.4)	17 (85.0)	15 (35.7)	7 (46.7)	15 (68.2)	7 (63.6)	41 (59.4)	120 (56.0)
Transgender	0 (0)	0 (0)	0 (0)	0 (0)	0 (0)	0 (0)	1 (1.5)	1 (0.5)
Age								
Range	0–9	24–43	14–18	24–38	24–61	25–63	20–63	0–63
Median	3	33	14	27	39	33.5	26	25
Hispanic (% per cohort)								
Yes	25 (71.4)	8 (40.0)	20 (47.6)	2 (13.3)	1 (4.5)	0 (0)	12 (17.4)	68 (31.8)
No	10 (28.6)	10 (50.0)	21 (50.0)	13 (86.7)	21(95.5)	11 (100)	57 (82.6)	143 (66.8)
Don't know	0 (0)	2 (10.10)	1 (2.4)	0 (0)	0 (0)	0 (0)	0 (0)	3 (1.4)
Race (% per cohort)								
White	3 (8.6)	4 (20.0)	1 (2.4)	9 (60.0)	18 (81.8)	5 (45.5)	37 (53.6)	77 (36.0)
African-American	3 (8.6)	2 (10.0)	21 (50.0)	4 (26.7)	0 (0)	2 (18.2)	4 (5.8)	36 (16.8)
Asian	3 (8.6)	3 (15.0)	2 (4.8)	1 (6.7)	3 (13.6)	3 (27.3)	21 (30.4)	36 (16.8)
American Indian	19 (54.2)	5 (25.0)	1 (2.4)	0 (0)	0 (0)	0 (0)	1 (1.5)	26 (12.1)
Other Pacific	0 (0)	0 (0)	1 (2.4)	0 (0)	0 (0)	0 (0)	0 (0)	1 (0.5)
Other or mixed	7 (20.0)	6 (30.0)	10 (23.8)	1 (6.7)	0 (0)	1 (9.0)	5 (7.2)	30 (14.0)
Don't know	0 (0)	0 (0)	6 (14.3)	0 (0)	1 (4.6)	0 (0)	1 (1.5)	8 (3.7)

### Specimen collection and analysis

Two nasopharyngeal samples per participant were collected on a weekly basis using minitip flocked swabs. Both samples were stored jointly in 2 ml DNA/RNA Shield (Zymo Research, Irvine, CA, USA) at 4–25 °C for up to 30 days and then stored at −80 °C in two aliquots. Nucleic acids were extracted from 200 µl of sample and 10 µl of internal control using the EasyMAG NucliSENS system (bioMerieux, Durham, NC, USA). Samples were then screened for viruses using the eSensor XT-8 respiratory viral panel (RVP; GenMark Dx, Carlsbad, CA, USA) [4], a multiplex PCR assay. The RVP system separately detects influenza A (any subtype, A/H1N1, A/H3N2, A/H1N1pdm2009) and B; RSV A and B; parainfluenza (PIV) 1, 2, 3 and 4; human metapneumovirus (HMPV); human rhinovirus (HRV); adenovirus B/E and C; and coronaviruses (CoV) 229E, NL63, OC43 and HKU1. Samples positive for a particular virus were identified by an electrical signal intensity of ⩾2 nA/mm^2^ (with the exception of CoV OC43 for which positive results were identified by an intensity of ⩾25 nA/mm^2^, per manufacturer specifications).

### Statistical analysis

We classified all specimens, irrespective of result, as symptomatic or asymptomatic according to the individual symptom score in the days surrounding the date of swab collection. We used multiple definitions as a standard for *symptomatic infection* does not exist. Definitions are summarised in Table 2: definitions 1–4 consider a time window of 7 days around the day of the collection, whereas definitions 5–8 use a window of 11 days. While some definitions use raw metrics, definitions 4 and 8 normalise scores by the average symptom score for each participant (average weekly total symptom scores for each participant ranged from 0 to 39). We refer to infections that do not satisfy one or more specified definitions as asymptomatic infections. The association between reporting respiratory symptoms and testing positive was calculated with the *χ*^2^ test. A ‘symptomatic week’ was defined as a calendar week where the total symptom score was ⩾10.

**Table 2.** Definitions of symptomatic infections

Def 1	At least 1 day in a −3/+3 days window presents daily score >3
Def 2	Minimum two symptoms within −3 and +3 days from the test, with at least one recorded as moderate or severe
Def 3	Total symptom score >9 in a −3/+3 days window
Def 4	Weekly score higher than two times the personal weekly average
Def 5	At least 1 day in a −3/+7 days window presents daily score >3
Def 6	Minimum two symptoms within −3 and +7 days from the test, with at least one recorded as moderate or severe
Def 7	Total symptom score >9 in a −3/+7 days window
Def 8	Weekly score higher than two times the personal 11 days average

Analyses were conducted using the total number of positive samples, as well as the number of illness-events. We defined an illness-event as a group of consecutive weekly swab specimens for a given individual that were positive for the same virus (allowing for a 1-week gap to account for false negatives and temporary low shedding).

The effects of different viruses and the severity of symptoms among different age groups were assessed using analysis of variance (ANOVA). The *χ*^2^ statistic was also used to assess pairwise differences. Participants were divided into four groups: children (under 10 years of age), teenagers (10–17 years of age), adults with daily contact with children (parents and pediatric ER doctors) and adults without daily contact with children. To assign adult participants to the correct category, we used information on household composition derived from the initial questionnaire.

In the analysis of symptoms by virus, specimens positive for more than one virus were excluded. To analyse the specific effects of different viruses, we grouped symptoms into *upper respiratory* symptoms (runny nose, sneezing, sore throat, watery eye), *lower respiratory* symptoms (cough, chest pain) and *systemic* symptoms (fever, chills, muscle pain).

## Results

Of the 4215 nasopharyngeal samples collected, 737 (17.5%) were positive for one or multiple respiratory viruses. Among the positive results, between 69% and 74% of the samples were classified as asymptomatic depending on the chosen definition (Table 2). Overall, 55% of positive specimens were asymptomatic by all definitions. Testing positive for one or more respiratory viruses was associated with an increased likelihood of being symptomatic (*P* < 0.0001); however, for the majority of symptomatic weeks (67%), RVP did not identify the presence of any respiratory virus. There was a weak association between pre-existing respiratory conditions (asthma, allergies) and the likelihood of experiencing symptomatic infections. However, the association was significant only for some definitions, and the effect on symptom score was marginally significant (*P* = 0.08 ANOVA).

Coinfections accounted for 10% of positive samples and were found most frequently among children; they occurred throughout the year and were predominantly a combination of HRV and adenovirus. Pairwise comparisons between single infections and coinfections across all eight definitions showed that testing positive for multiple viruses was not associated with more severe symptoms.

**Fig. 1.**
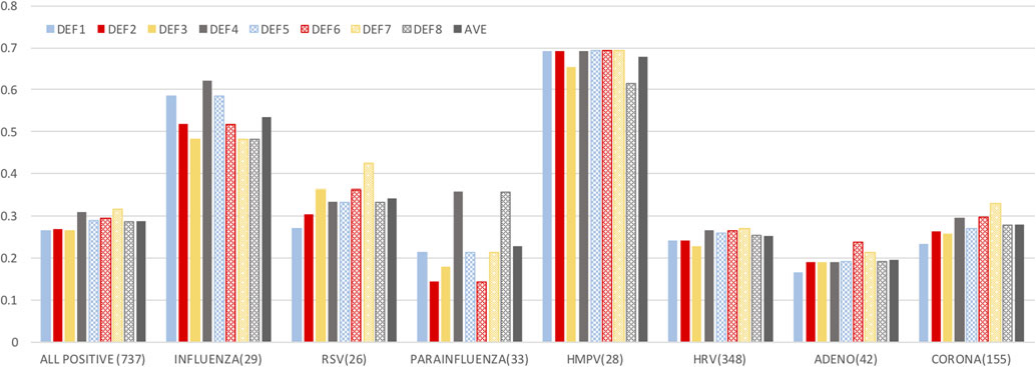
Symptoms by virus. The bars show the fraction of positive results associated with a symptomatic definition (Table 2). The total number of infections with each virus is reported (co-infections are excluded from the individual virus counts but are included in the *all positive* category). Same colour/different filling bars pair corresponding definitions that span different time windows.

Among the viruses considered, influenza and HMPV were associated with significantly higher symptom scores than RSV, HRV, CoV, adenovirus and PIV. As shown in Figure 1, more than 50% of influenza and HMPV infections were classified as symptomatic by a majority of definitions, whereas the other viruses were mostly asymptomatic according to all definitions (*P* *<* *0*.01 and *P* < 0.001 when comparing, respectively, the ratio of symptomatic influenza and HMPV infections to the other viral infections).

**Fig. 2.**
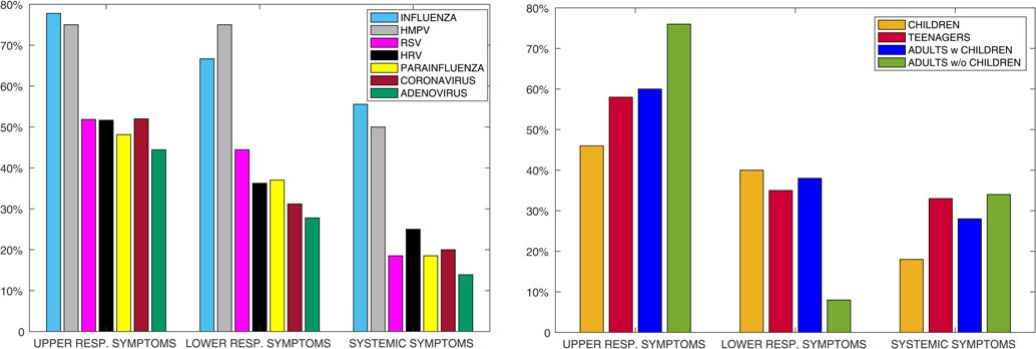
Specific symptoms per (a) virus and (b) age group. Here infection events (not positivity counts) are analysed. A viral event is considered positive for upper respiratory, lower respiratory or systemic symptoms if the individual reported at least one of the characterizing symptoms during the 7 days surrounding the test date.

The analysis of specific symptoms confirmed the higher severity of influenza and HMPV for lower, upper and systemic symptoms (Fig. 2a). Upper respiratory symptoms were most commonly associated with positive results for all viruses and for all age groups, followed by lower respiratory symptoms and then systemic symptoms (Fig. 2a and b). Infected children showed a similar percentage of upper and lower respiratory symptoms, and teenagers showed a similar percentage of lower respiratory and systemic symptoms. The majority of HMPV infections presented with both upper and lower respiratory symptoms.

**Fig. 3.**
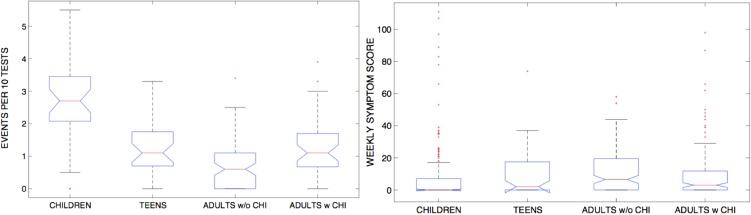
Distribution of number of illness events (a) and associated symptoms score (b) across age groups. Symptom score was computed for ±3 days around the date of sample collection.

Higher severity of symptoms was not associated with higher frequency of infection. Figure 3 shows that while children were most frequently infected with a respiratory virus (they presented with the highest number of viral shedding events per season), they recorded (as reported by their parents) the lowest symptom scores on average. Adults without daily contact with children reported the highest symptom score (the difference was significant only between children and adults without contacts with children, *P* = 0.003). This finding holds when controlling for length of infection, as longer-lasting infections were more frequent in children.

## Discussion

Host response to respiratory viruses is heterogeneous: some presumed infections, measured by detectable shedding of virus, exhibit no symptoms or signs of disease, whereas others result in more serious symptoms. When assessing the burden of an infectious disease, it is important to evaluate both the prevalence of the infection within the population and the range of its symptomatic manifestations. We have already shown in previous studies that respiratory viral infections are widespread within the community throughout the year [11–13]. In [11] we showed that most individuals, particularly children and their close contacts, contract multiple respiratory infections per year. Here, we analysed the symptoms reported by the same infected individuals and characterised them by virus and by age group.

The presence of respiratory symptoms was associated with testing positive for one or more respiratory viruses. However, the majority of symptomatic manifestations were not paired with a positive RVP result. The origin of these symptomatic, negative RVP results could be due to allergies, bacterial infections or potential viral infections with pathogens that have not yet been identified or for which the viral panel does not test.

One of the main goals of this analysis was to estimate the *asymptomatic ratio*, that is, the fraction of infections occurring without symptoms. The *asymptomatic ratio* is an important indicator for constraining both true respiratory virus prevalence within a community and the potential for further disease transmission [14]. Asymptomatic carriers can, in fact, contribute to disease spread by generating (possibly symptomatic) secondary infections. Estimates of the asymptomatic ratio vary widely not only across diseases (from 95% of polio virus infections to <10% of measles), but also within the same disease due to different diagnostic testing procedures (PCR *vs.* serological tests [15]) and sampling approaches (household *vs.* longitudinal studies).

Among respiratory viruses, the role of asymptomatic infection is poorly understood. For influenza alone, the prevalence of asymptomatic infection has been estimated to be as low as 9.4% and as high as 90% depending on the virus type, study, season and definition of asymptomatic infection [15, 16]. There is also some evidence that viral shedding correlates with symptom severity and that the contagiousness of asymptomatic individuals is less than for symptomatic persons [17,18]. The rationale is that respiratory symptoms (coughing, sneezing, runny nose) help spread pathogens through droplet transmission, either inhaled or settled [19]. However, the absence of symptoms might bring asymptomatic infected individuals into greater contact with susceptible persons outside the home. This is particularly true for infants and toddlers whose behaviour, hygiene habits and close physical contact typically favour the spread of germs, especially in childcare settings. Whether greater contact occurs and makes asymptomatic individuals effectively as contagious as symptomatic persons is not known. An additional difficulty is that a standard, accepted definition of *symptomatic infection* does not exist. Further, perception of symptoms is highly subjective, and it may be difficult to assess whether a symptom is caused by the pathogen. For example, chronic symptoms can occur – runny nose is a common sign in children that does not necessary imply viral infection – and allergies can cause sneezing. Fever, muscle pain and chills may also be caused by infections other than respiratory viruses. For these reasons, we adopted multiple definitions of ‘symptomatic episodes’, including personalised metrics.

More than half of the RSV, adenovirus, PIV, CoV and HRV infections documented here were asymptomatic according to all definitions. Influenza and HMPV infections caused significantly more symptoms than these other viruses; however, given its high prevalence, HRV was responsible for more than half of symptomatic episodes. In general, all viral infections presented with similar symptoms, with upper respiratory manifestations being more frequent, whereas systemic symptoms such as fever, muscle pain and chills were less typical. For surveillance in which ILI (defined as fever plus cough and/or sore throat) is used to record respiratory infections among people seeking care, this constraint may mean that many viral infections go undetected. Consistent with previous findings [20, 21] we did not find an association between viral co-infection and the likelihood of being symptomatic or presenting with more severe symptoms. Regardless of the definition, our findings underscore the extremely high proportion of respiratory viral infections that are asymptomatic. Further analysis is required to capture the role played by asymptomatic individuals in outbreak transmission dynamics, specifically the relative infectiousness of asymptomatic *vs.* symptomatic infections.

There was considerable heterogeneity in individual immune response: some individuals seemed to be consistently less predisposed to developing respiratory symptoms upon viral infection, whereas others were always symptomatic when testing positive. Infections in children were less symptomatic than in adults, even though children proved to be more frequently infected with respiratory viruses [11]. Frequent infections may enhance the ability of the immune system to identify and respond to pathogens; hence, the group subjected to the fewest respiratory viral infections (adults without children) reported the highest average symptom scores. However, the apparent lower pathogenicity among children may simply be an artefact introduced due to parents reporting symptoms for their children. Some symptoms, such as sore throat, muscle pain and chest pain, are difficult to identify in young children, and in fact they were less reported in this age category than among adults and teenagers. This possible bias in the reporting of symptoms is one limitation of our study. The daycare and workplace connections among some individuals in our cohort are another possible bias: some infections were likely caused by the same strain; thus the symptom profiles could be due to specific features of the pathogen rather than individual immune response. Further, all the children in our cohort were attending daycare or were siblings of children attending daycare. Interactions within the daycare setting could be a confounder in this analysis. Future studies should investigate the genetic basis of heterogeneity in host response to respiratory virus infection in order to identify the regulatory pathways controlling reactions to these infections. Moreover, longitudinal studies of this type, involving large networks of connected individuals, are needed to assess the role of asymptomatic infections in the transmission of respiratory viruses.
